# Vibrational spectroscopy of metal methanesulfonates: M = Na, Cs, Cu, Ag, Cd

**DOI:** 10.1098/rsos.171574

**Published:** 2018-04-18

**Authors:** Stewart F. Parker, Lisha Zhong

**Affiliations:** ISIS Facility, STFC Rutherford Appleton Laboratory, Chilton, Didcot, Oxon OX11 0QX, UK

**Keywords:** methanesulfonate, inelastic neutron scattering spectroscopy, infrared spectroscopy, Raman spectroscopy, DFT

## Abstract

In this work, we have used a combination of vibrational spectroscopy (infrared, Raman and inelastic neutron scattering) and periodic density functional theory to investigate six metal methanesulfonate compounds that exhibit four different modes of complexation of the methanesulfonate ion: ionic, monodentate, bidentate and pentadentate. We found that the transition energies of the modes associated with the methyl group (C–H stretches and deformations, methyl rock and torsion) are essentially independent of the mode of coordination. The SO_3_ modes in the Raman spectra also show little variation. In the infrared spectra, there is a clear distinction between ionic (i.e. not coordinated) and coordinated forms of the methanesulfonate ion. This is manifested as a splitting of the asymmetric S–O stretch modes of the SO_3_ moiety. Unfortunately, no further differentiation between the various modes of coordination: unidentate, bidentate etc … is possible with the compounds examined. While it is likely that such a distinction could be made, this will require a much larger dataset of compounds for which both structural and spectroscopic data are available than that available here.

## 1. Introduction

**Figure 1.**
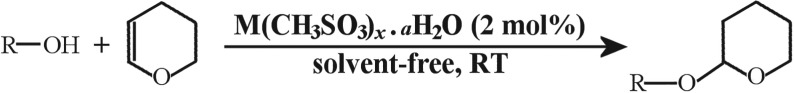
Tetrahydropyranylation of an alcohol catalysed by a metal methanesulfonate.

Metal methanesulfonates (*M*(CH_3_SO_3_)*x* · *n*H_2_O) are compounds of interest because of the role they play as catalysts for numerous reactions in organic synthesis including (but not limited to) Mannich, Biginelli reaction, esterification and tetrahydropyranylation of alcohols and phenols [1] (figure 1). This is due to their low toxicity, low cost and low reactivity with air and water [2].

Although these substances are rarely found naturally, some forms can be found in the sea as a result of methanesulfonic acid (CH_3_SO_3_H) being produced and emitted by marine phytoplankton (via the oxidation of dimethyl sulfide). This then undergoes chemical reactions with the cations present in sea salt e.g. Mg^2+^, K^+^, Ca^2+^ and Na^+^, to form their respective metal methanesulfonate salts [3,4]. The natural methods through which these salts form mean that they are vital to exploring the past due to nature of their deposition in ice cores, with a particular focus on Mg(CH_3_SO_3_)_2_· *n*H_2_O and Na(CH_3_SO_3_)_2_· *n*H_2_O—where the sodium salt is the most abundant [5]. It is worth noting, though, that the magnesium salt found is restricted to the Last Glacial Maximum ice which forms during the coldest period in the glacial cycle. Only during this time did the atmospheric conditions enable preservation of the salt because normally when Mg(CH_3_SO_3_)_2_· *n*H_2_O is deposited, it subsequently dissolves in acidic ice [3].

Metal methanesulfonate salts have also recently come into use in electrophysiological research as a common substance used for patch-clamp techniques to allow for the study of ion channels in cells. Caesium methanesulfonate is especially appropriate for this role as Cs^+^ can block K^+^ currents [6] while CH_3_SO_3_^−^ can block Cl^−^ channels [7]. The implications from this technique are far ranging, as it can be used to investigate many biological processes from canine atrial cells [6] to hippocampal pyramidal cells in young rats [8].

The vibrational spectra of metal methanesulfonate salts have been investigated several times [9–13]. In the present work, we use a combination of infrared, Raman and inelastic neutron scattering (INS) spectroscopies to observe all of the modes of the methanesulfonate ion in both ionic (M = Na, Cs) and complexed (M = Cu, Ag, Cd) environments. The assignments are supported by periodic density functional theory (DFT) calculations of the Na, Cs and Cu salts.

## 2. Experimental

### 2.1. Materials

The M(CH_3_SO_3_) salts, M = Na, Cs, Ag, were purchased from Aldrich and used as received, except for the Ag salt which was dried overnight at 110°C in a vacuum oven, to remove traces of adsorbed water. The Cu(H_2_O)_4_(CH_3_SO_3_)_2_, Cu(D_2_O)_4_(CH_3_SO_3_)_2_, Cd(H_2_O)_2_(CH_3_SO_3_)_2_, Cd(D_2_O)_2_(CH_3_SO_3_)_2_ and a sample of Ag(CH_3_SO_3_) were kindly provided by Durham University. These were in sealed quartz ‘lollipops’. After the Raman and INS measurements were made, the lollipops were broken open in order to measure the infrared spectra. Powder X-ray diffraction confirmed the identities of the samples. In further confirmation, the data reported here are for the Ag salt from Aldrich; however, comparison with the spectra of the Ag salt of the Durham sample showed them to be identical.

### 2.2. Vibrational spectroscopy

INS spectra were recorded at less than 20 K using TOSCA [14] at ISIS (http://www.isis.stfc.ac.uk/). The spectra are available at the INS database: http://wwwisis2.isis.rl.ac.uk/INSdatabase/. Infrared spectra were recorded using a Bruker Vertex70 FTIR spectrometer, over the range 100 to 4000 cm^−1^ at 4 cm^−1^ resolution with a DLaTGS detector using 64 scans and the Bruker Diamond ATR. The use of the ultra-wide range beamsplitter enabled the entire spectral range to be recorded without the need to change beamsplitters. The spectra have been corrected for the wavelength-dependent variation in pathlength using the Bruker software. FT-Raman spectra were recorded with a Bruker MultiRam spectrometer using 1064 nm excitation, 4 cm^−1^ resolution, 500 mW laser power and 64 scans. Dispersive Raman spectra were recorded with a Bruker Senterra Raman spectrometer using 532 nm excitation. All the infrared and Raman spectra were measured in air at room temperature. The Raman spectra of the Cu complex have been baseline corrected to remove a broad fluorescence background.

### 2.3. Computational studies

The plane wave pseudopotential-based program CASTEP was used for the calculation of the vibrational transition energies and their intensities [15,16]. The generalized gradient approximation Perdew–Burke–Ernzerhof functional was used in conjunction with optimized norm-conserving pseudopotentials. In the electronic supplementary material, table S1 gives the details of the calculations. All of the calculations were converged to better than |0.0035| eV Å^−1^. After geometry optimization, the vibrational spectra were calculated in the harmonic approximation using density functional perturbation theory [17]. This procedure generates the vibrational eigenvalues and eigenvectors, which allows visualization of the modes within Materials Studio (http://accelrys.com/products/collaborative-science/biovia-materials-studio/) and is also the information needed to calculate the INS spectrum using either the programs ACLIMAX [18] or AbINS [19]. We emphasize that the transition energies have *not* been scaled.

## 3. Results and discussion

In this section, we will first provide a complete assignment for Cs(CH_3_SO_3_) based on a periodic DFT calculation. We will then use this to assign the internal modes of the methanesulfonate ion in the other materials.

### 3.1. Cs(CH_3_SO_3_)

**Figure 2.**
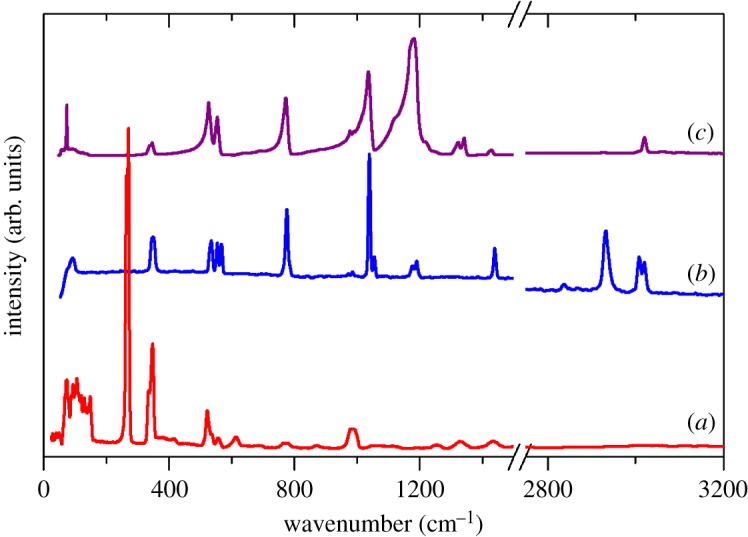
(*a*) INS, (*b*) FT-Raman and (*c*) infrared spectra of Cs(CH_3_SO_3_).

Figure 2 shows the INS, Raman and infrared spectra of Cs(CH_3_SO_3_). Table 1 lists the transition energies and assignments, which are based on visualization of the modes from the CASTEP calculation. In the electronic supplementary material, table S2 lists all of the calculated modes with their assignments. Cs(CH_3_SO_3_) crystallizes in the orthorhombic space group *P*nma (no. 62) with four formula units in the primitive cell [20], thus there are 108 modes in total comprising 3 acoustic modes, 21 optic translational modes of the ions, together with 12 librational and 72 internal modes of the methanesulfonate ion. A complete factor group analysis is given in the electronic supplementary material, table S2. The methanesulfonate ion is on a general position, however it has *C*_3v_ symmetry within the accuracy of the analysis. Assuming *C*_3v_ symmetry, the 18 internal modes of the isolated methanesulfonate ion can be classified as: C–H stretch (*A*_1_ + *E*), H–C–H bend (*A*_1_ + *E*), methyl rock (*E*), S = O stretch (*A*_1_ + *E*), O = S = O bend (*A*_1_ + *E*), C–S stretch (*A*_1_), sulfonate rock (*E*) and C–S torsion (*A*_2_).

**Table 1.** Transition energies (cm^−1^) of the internal modes of the methanesulfonate ion in the Na and Cs compounds. sh, shoulder; modes in italics are overtones or combinations.

Na			Cs			
INS	Raman	infrared	INS	Raman	infrared	description
	3023, 3005	3018, 3004		3020, 3006	3021	CH_3_ asymmetric stretch
	2939	2941		2942, 2931		CH_3_ symmetric stretch
	2863, 2829			2838		*2 × CH_3_ symmetric bend*
1432, 1400	1453, 1435, 1413	1451, 1434, 1415	1434	1438	1430, 1421sh	CH_3_ asymmetric bend
1325		1352, 1336	1330		1342, 1323	CH_3_ symmetric bend
1183	1218, 1204, 1195, 1169	1247, 1215sh, 1183		1191, 1176	1219sh, 1179, 1115sh	SO_3_ asymmetric stretch
1049	1078, 1061sh, 1052	1074sh, 1061, 1054		1055, 1036	1036	SO_3_ symmetric stretch
981, 968	992sh, 986, 972, 960sh	990, 965	991, 980	986, 972	988, 979	CH_3_ rock
784	799, 792sh 781	787, 781	780, 763	775	771	C–S stretch + SO_3_ symmetric bend
699, 644, 607			613			2 × SO_3_ rock C–S torsion (307 cm^−1^) + SO_3_ rock 2 × C–S torsion (307 cm^−1^)
567, 562, 552	578, 568sh, 560	574sh, 559,	564, 556sh	568, 552	553	SO_3_ symmetric bend + C–S stretch
531	544, 532	544, 534, 527sh	536, 521	533	527	SO_3_ asymmetric bend
425						*2 × C–S torsion (220 cm^−1^)*
350, 336	354	351	347, 334	351, 345sh	346, 337sh	SO_3_ rock
309, 294, 270, 224, 217			271, 264			C–S torsion

For a centrosymmetric molecule in the gas phase the rule of mutual exclusion (no coincidences in the infrared and Raman spectra) is strictly valid. In the solid state, this is only rigorously true for a centrosymmetric molecule in a crystal that crystallizes in a centrosymmetric system and has only one molecule in the primitive cell. A classic example is K_2_[PtCl_6_], which crystallizes in a cubic space group and the ${\left[\text{PtC}{\text{l}}_{6}\right]}^{2-}$ ion occupies an octahedral site [21]. For a centrosymmetric crystal with two molecules in the unit cell, the vibrations form in-phase and out-of-phase pairs; this is the factor group splitting and arises from interactions between the molecules. In the limit that the interaction is zero, the in-phase and out-of-phase pairs are accidentally degenerate. If one mode is Raman active and the other is infrared active, then even though it is a centrosymmetric system, the modes will occur at the same transition energy in both spectra. It is the degree of coupling between the species that determines the difference in the transition energies in the two forms of spectroscopy. This is explored in more detail in the electronic supplementary material, figure S1 and table S2. In the present case, although the structure is centrosymmetric, the factor group splitting is small, even though there are four molecules in the unit cell, meaning that modes occur at similar energies in both sets of spectra. There is no evidence for symmetry-breaking in this system, as has been found for other alkali metal compounds [22,23].

The spectra illustrate the complementarity of the three techniques. INS has no symmetry-based selection rules [24], however there is a strong ‘propensity’ rule that motions that involve displacement of hydrogen dominate the spectrum. This is dramatically shown by the strongest mode in the INS spectrum at approximately 270 cm^−1^, which is assigned to the torsion about the C–S bond. The computational study (electronic supplementary material, table S4) confirms that this mode has essentially zero intensity in both the infrared and Raman spectrum, but as a consequence of the large amplitude hydrogen motion, it is very strong in the INS spectrum. It is also notable that only the methyl modes (torsion, rock, deformations) have significant intensity in the INS spectrum. This demonstrates that the coupling between the CH_3_ and SO_3_ moieties in the ion is weak. By contrast, the strongest modes in the infrared and Raman spectra are motions of the SO_3_ moiety.

**Figure 3.**
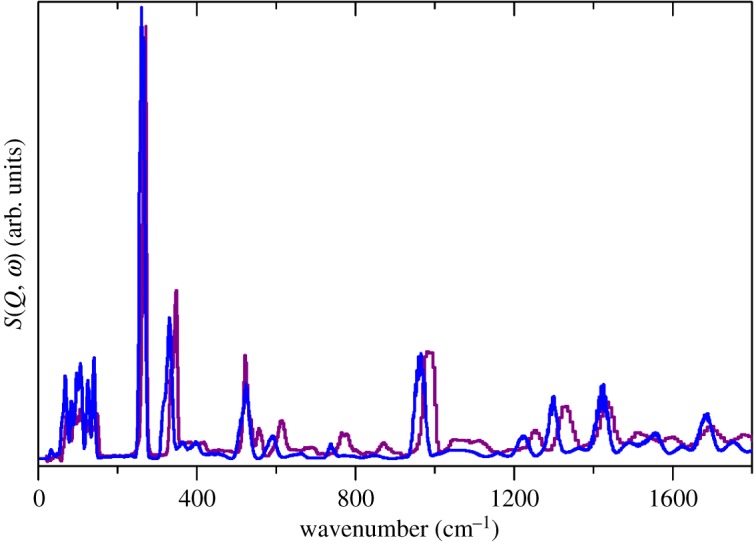
Comparison of experimental (purple) and calculated (blue) INS spectra of Cs(CH_3_SO_3_).

Figures 3, and Figures S2 and S3 in the electronic supplementary material compare the observed and calculated INS, Raman and infrared spectra of Cs(CH_3_SO_3_). It can be seen that for the INS and Raman spectra the agreement is almost quantitative in terms of both transition energy and relative intensity. For the infrared spectrum, the intensity agreement is poorer, which is a consequence of the use of the same band width for all of the calculated modes; inspection of the experimental spectrum shows that this is not the case. Nonetheless, it is clear that the calculation has provided a reliable basis for the spectral assignment and this will be used for the other salts.

### 3.2. Na(CH_3_SO_3_)

**Figure 4.**
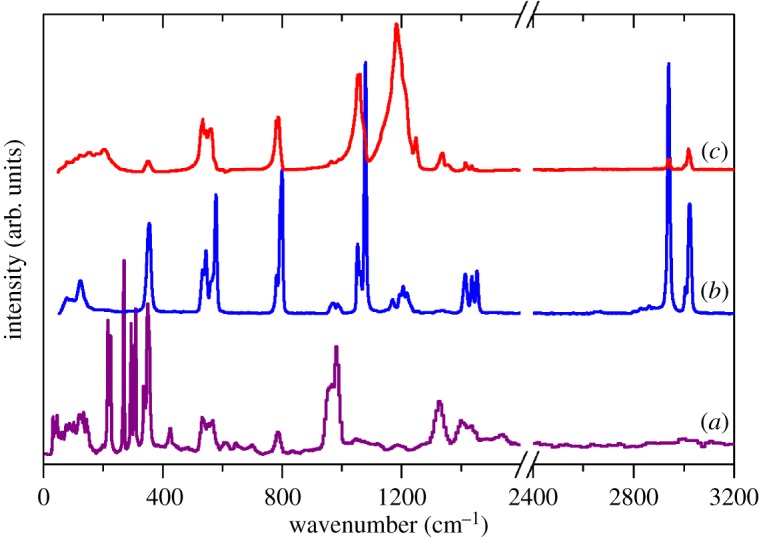
(*a*) INS, (*b*) FT-Raman and (*c*) infrared spectra of Na(CH_3_SO_3_).

**Figure 5.**
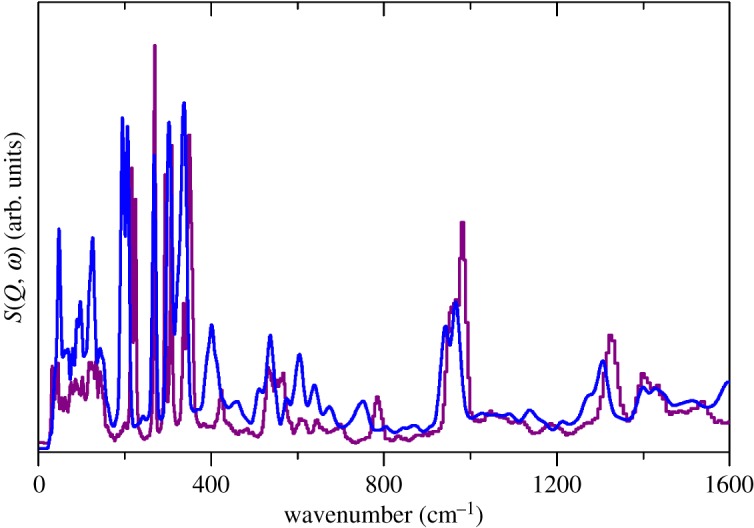
Comparison of experimental (purple) and calculated (blue) INS spectra of Na(CH_3_SO_3_).

Figure 4 shows the INS, Raman and infrared spectra of Na(CH_3_SO_3_). The similarities to those of the Cs salt are striking, except for the greater intricacy of the Na spectra. The structure of the Na salt is complex with 20 formula units in the primitive cell [25]. The very large number of optic modes (537) means that it is not possible to calculate the Raman spectrum in a reasonable time. However, as figure 5 shows, the calculated INS spectrum is in excellent agreement with the experimental spectrum. In the electronic supplementary material, figure S4 compares the observed and calculated infrared spectra. Table 1 lists the observed bands and their assignments.

### 3.3. Cu, Cd and Ag methanesulfonates

**Figure 6.**
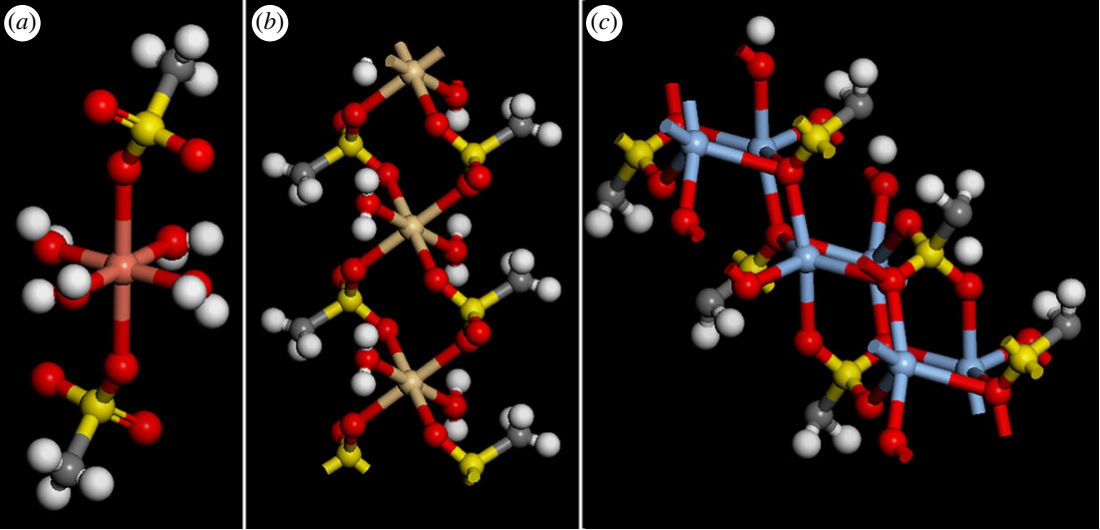
Structure of: (*a*) Cu(H_2_O)_4_(CH_3_SO_3_)_2_, (*b*) Cd(H_2_O)_2_(CH_3_SO_3_) and (*c*) Ag(CH_3_SO_3_). (Colour key: white, H; grey, C; red, O; yellow, S; copper, Cu, brown, Cd; blue, Ag).

In contrast to the simple ionic bonding present in the alkali metal salts, the structures of the Cu [26], Cd [27] and Ag [28] compounds are more diverse and more complex. The Cu compound has square planar [Cu(H_2_O)_4_]^2−^ ions with the apical positions occupied by an oxygen atom of the methanesulfonate ion. Each methanesulfonate ion is only coordinated to one [Cu(H_2_O)_4_]^2−^ ion. For the Cd compound, the Cd atom is located at the inversion centre of an octahedron with the O atoms of two water molecules at the apices and the O atoms belonging to four methanesulfonato groups in the horizontal plane. The Cd atoms are bridged by the methanesulfonato groups, so forming parallel infinite chains running along the *b* axis. The Ag complex has an even more elaborate structure. There is no distinct molecule; the methanesulfonato groups act as pentacoordinating ligands. Thus, each Ag atom is at the centre of a very distorted trigonal bipyramid. Figure 6 shows the three structures, which exhibit monodentate, bidentate and pentadentate coordination, respectively, of the methanesulfonato ligand.

**Figure 7.**
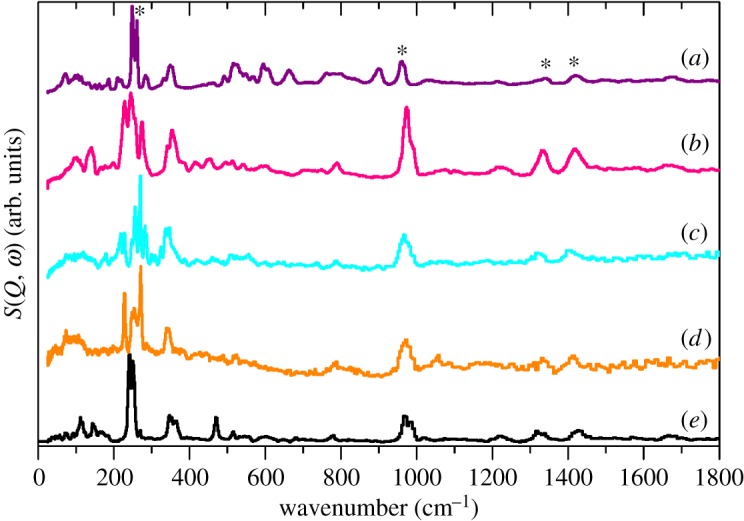
INS spectra of: (*a*) Cu(H_2_O)_4_(CH_3_SO_3_)_2_, (*b*) Cu(D_2_O)_4_(CH_3_SO_3_)_2_, (*c*) Cd(H_2_O)_2_(CH_3_SO_3_)_2_, (*d*) Cd(D_2_O)_2_(CH_3_SO_3_)_2_ and (*e*) Ag(CH_3_SO_3_). Methyl modes (torsion, rock, deformations) are indicated by asterisks.

The INS spectra of the Cu, Cd and Ag materials (including the D_2_O isotopomers for Cu and Cd) are shown in figure 7. As with the alkali metal salts (figures 3 and 5 and table 1) the features assigned to modes of the methyl group dominate the spectra and show a remarkable constancy in position (table 2). This is consistent with the methyl groups not being involved in the bonding; in all three examples they project into vacant space in the structure. The modes due to the coordinated water molecules in the Cu and Cd compounds give rise to librational modes in the 400–900 cm^−1^ region [29–31]. These are relatively weak and are readily assigned because they are shifted and much weaker in the D_2_O-containing samples.

**Table 2.** Transition energies (cm^−1^) of the internal modes of the methanesulfonate ion in the Cu, Cd and Ag compounds. sh, shoulder; modes in italics are overtones or combinations.

Cu			Cd			Ag			
INS	Raman	infrared	INS	Raman	infrared	INS	Raman	infrared	description
	3029, 3012	3029, 3012		3037, 3025	3040		3031, 3021	3031, 3019	CH_3_ asymmetric stretch
	2940	2940		2945	2950		2940	2939	CH_3_ symmetric stretch
1421	1422	1428, 1414	1405	1422, 1414sh	1428, 1415	1426	1430, 1421	1434, 1415	CH_3_ asymmetric bend
1342		1339	1323		1332, 1322sh	1328		1338, 1319	CH_3_ symmetric bend
	1207	1216, 1138		1217, 1198sh	1241, 1155		1200	1168, 1116	SO_3_ asymmetric stretch
1215			1187			1219			*C–S torsion + CH_3_ rock*
	1123			1130			1129		*C–S stretch + SO_3_ rock*
	1045	1043		1065	1056		1050	1014	SO_3_ symmetric stretch
963	964	966, 957	968	1002, 991, 969	990sh, 975,	993, 974	961	986, 967	CH_3_ rock
902	896		897		893				H_2_O libration
801	791	776	787	792	797sh, 785	779	784	771	C–S stretch + SO_3_ symmetric bend
605, 593	601	634, 586			658, 616				H_2_O libration
568, 549, 536sh, 525, 516	551, 528	544, 515	566, 555, 545, 536, 523, 509	573, 541, 519	571, 549, 539, 509		560, 533, 521	544, 526, 515	SO_3_ symmetric bend + C–S stretch
491	488		486, 474, 458		488				
464, 453, 438	436		418		449, 440, 424, 415				
357sh, 347	353	348	347, 338	390, 351	346	365, 347		353	SO_3_ rock
261, 248			270, 254, 246		256, 247	250, 241			C–S torsion

**Figure 8.**
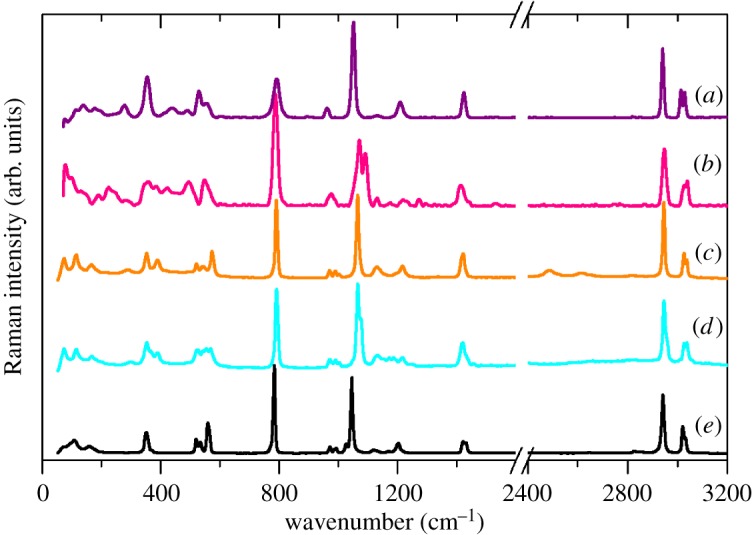
Raman spectra of: (*a*) Cu(H_2_O)_4_(CH_3_SO_3_)_2_, (*b*) Cu(D_2_O)_4_(CH_3_SO_3_)_2_, (*c*) Cd(H_2_O)_2_(CH_3_SO_3_)_2_, (*d*) Cd(D_2_O)_2_(CH_3_SO_3_)_2_ and (*e*) Ag(CH_3_SO_3_).

The Raman spectra are shown in figure 8. Again, as with the alkali metal salts, the spectra are dominated by SO_3_ modes. Only the S–O symmetric stretch at approximately 1050 cm^−1^ shows a small variation in the complexes.

**Figure 9.**
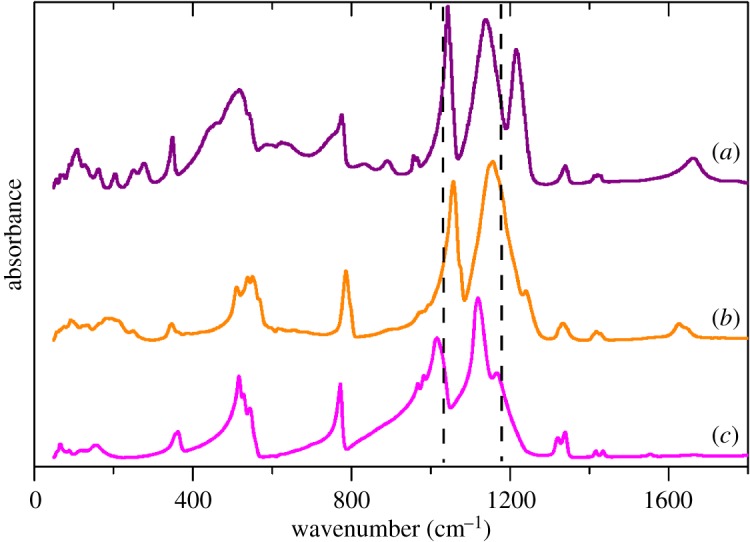
Infrared spectra of: (*a*) Cu(H_2_O)_4_(CH_3_SO_3_)_2_, (*b*) Cd(H_2_O)_2_(CH_3_SO_3_)_2_ and (*c*) Ag(CH_3_SO_3_). The dashed lines show the transition energies of the symmetric (left) and asymmetric (right) S–O stretch modes in Cs(CH_3_SO_3_).

The infrared spectra of Cu(H_2_O)_4_(CH_3_SO_3_)_2_, Cd(H_2_O)_2_(CH_3_SO_3_)_2_ and Ag(CH_3_SO_3_) in the fingerprint region are shown in figure 9. (The complete 0–4000 cm^−1^ spectra of these compounds and those of Cu(D_2_O)_4_(CH_3_SO_3_)_2_ and Cd(D_2_O)_2_(CH_3_SO_3_)_2_ are shown in the electronic supplementary material, figure S5). For the alkali metal salts, the spectra are consistent with local *C*_3v_ symmetry, the degenerate modes only show a small or no splitting. This is not the case here; the asymmetric S–O stretch mode is both strongly perturbed and is downshifted with respect to the M = Na and Cs salts. It can be seen that the degeneracy of the S–O asymmetric stretch is lifted and two modes appear. (For the Cd salt, this manifests as a broadening of the band; compare the width of the symmetric and asymmetric S–O stretches.) Unfortunately, there is no apparent correlation between the degree of splitting and the type of coordination as there is, for example, with carbonate complexes [32].

## 4. Conclusion

At the outset of this work, it was hoped that the mode of coordination of the methanesulfonate ion would show characteristic patterns in the vibrational spectra. These could then be used as a fingerprint for the type of coordination, as found for other ions, e.g. nitrite, sulfate and carbonate [32]. We have studied six compounds that exhibit four different modes of coordination. We found that the transition energies of the modes associated with the methyl group (C–H stretches and deformations, methyl rock and torsion) are essentially independent of the mode of coordination. The SO_3_ modes in the Raman spectra also show little variation. In the infrared spectra, there is a clear distinction between ionic (i.e. not coordinated) and coordinated forms of the methanesulfonate ion. This is manifested as a splitting of the asymmetric S–O stretch modes of the SO_3_ moiety. Unfortunately, no further differentiation between the various modes of coordination, unidentate, bidentate etc. … , is possible with the compounds examined. While it is likely that such a distinction could be made, this will require a much larger dataset of compounds for which both structural and spectroscopic data is available than that used here.

## Supplementary Material

Table of calculated transition energies for Cs(CH3SO3) and comparison of observed and calculated spectra and complete spectra of the compounds.

## Data accessibility

The data which underpin this work are available via TopCat, the ISIS Facility's open access online data repository at: https://data.isis.stfc.ac.uk/doi/investigation/88613665.
