# Supplementary material for: Vibrational spectroscopy of metal methanesulfonates: M = Na, Cs, Cu, Ag, Cd
Source: R Soc Open Sci. 2018 Apr 18;5(4):171574. doi: 10.1098/rsos.171574 (PMC5936901; doi:10.1098/rsos.171574)
Supplement: Table of calculated transition energies for Cs(CH3SO3) and comparison of observed and calculated spectra and complete spectra of the compounds. [file rsos171574supp1.docx]

**Vibrational spectroscopy of metal methanesulfonates:
M = Na, Cs, Cu, Ag, Cd**

Stewart F. Parker* and Lisha Zhong

*ISIS Facility, STFC Rutherford Appleton Laboratory, Chilton, Didcot, Oxon OX11 0QX, UK,*

Author for correspondence:
Stewart F. Parker
e-mail: [stewart.parker@stfc.ac.uk](mailto:stewart.parker@stfc.ac.uk)
ORCiD: SFP 0000-0002-3228-2570
ORCiD: LZ 0000-0003-4966-0408

**Electronic Supplementary Material**

**Table of Contents**

| Title page and detailed list of contents of the Electronic Supporting Information. | 1 |
| --- | --- |
| **Table S1** Computational details for the metal methanesulfonates | 2 |
| Factor group analysis for Cs(CH_3_SO_3_) and **Table S2** | 3 |
| Factor group splitting in a centrosymmetric system, **Figure S1** and **Table S3** | 4 |
| **Table S4** Calculated modes with their assignments for Cs(CH_3_SO_3_) | 5 |
| **Figure S2.** Comparison of experimental and calculated Raman spectra of Cs(CH_3_SO_3_) | 12 |
| **Figure S3.** Comparison of experimental and calculated infrared spectra of Cs(CH_3_SO_3_). | 12 |
| **Figure S4.** Comparison of experimental and calculated INS spectra of Na(CH_3_SO_3_). | 13 |
| **Figure S5.** Infrared spectra, 0 – 4000 cm^-1^, of: (a) Cu(H_2_O)_4_(CH_3_SO_3_)_2_,  (b) Cu(D_2_O)_4_(CH_3_SO_3_)_2_, (c) Cd(H_2_O)_2_(CH_3_SO_3_)_2_, (d) Cd(D_2_O)_2_(CH_3_SO_3_)_2_ and  (e) Ag(CH_3_SO_3_). | 13 |
| **References** | 13 |

**Table S1** Computational details for the metal methanesulfonates.

| Metal | Spin polarised? | Plane wave cutoff / eV | Monkhorst-Pack grid | Number of k-points |
| --- | --- | --- | --- | --- |
| Na | No | 830 | 4×4×6 | 12 |
| Cs | No | 830 | 4×6×4 | 12 |
| Cu | Yes | 880 | 4×4×6 | 24 |
| Cd | No | 830 | 4×4×6 | 48 |
| Ag | No | 850 | 4×6×6 | 36 |

**Factor group analysis of Cs(CH_3_SO_3_).**

Cs(CH_3_SO_3_) crystallises in the orthorhombic space group *P*nma ≡ $D_{2h}^{16}$ (no. 62) with four formula units in the primitive cell [1], thus there are 108 modes in total comprising 3 acoustic modes, 21 optic translational modes of the ions, together with 12 librational and 72 internal modes of the methanesulfonate ion. The C, S and one of each of the O and H atoms lie on special positions (Wyckoff *c*, point group *C*_s_), with the remaining O and H atoms are on general sites. Thus the [CH_3_SO_3_]^-^ ion has a crystallographically imposed mirror plane, however, the [CH_3_SO_3_]^-^ ion has *C*_3v_ symmetry to within the error of the structural determination. The Cs^+^ ion is also on a Wyckoff *c* site. Using the correlation method [2], the factor group analysis of Cs(CH_3_SO_3_) is given in Table S2. All *gerade* modes are Raman active, *B*_1u_, *B*_2u_, *B*_3u_ are infrared active and *A*_u_ is inactive in both forms of spectroscopy. Note that *all* modes are allowed in the INS spectrum, although those involving predominantly the sulphonate group will be very weak.

**Table S2** Factor group analysis of Cs(CH_3_SO_3_).

|  |  | *C*_s_ site species | correlate *via* *zx* | *D*_2h_ factor group species |
| --- | --- | --- | --- | --- |
| Cs^+^ trans |  |  |  |  |
| 4 | 2 | A′ |  | 2*A*_g_, 2*B*_2g_, 2*B*_1u_, 2*B*_3u_ |
| 4 | 1 | A″ |  | *B*_1g_, *B*_3g_, *A*_u_, *B*_2u_ |
|  |  |  |  |  |
| [CH_3_SO_3_]^-^ trans |  |  |  |  |
| 4 | 2 | A′ |  | 2*A*_g_, 2*B*_2g_, 2*B*_1u_, 2*B*_3u_ |
| 4 | 1 | A″ |  | *B*_1g_, *B*_3g_, *A*_u_, *B*_2u_ |
|  |  |  |  |  |
| [CH_3_SO_3_]^-^ lib |  |  |  |  |
| 4 | 1 | A′ |  | *A*_g_, *B*_2g_, *B*_1u_, *B*_3u_ |
| 4 | 2 | A″ |  | 2*B*_1g_, 2*B*_3g_, 2*A*_u_, 2*B*_2u_ |
|  |  |  |  |  |
| [CH_3_SO_3_]^-^ vib |  |  |  |  |
| 4 C–H stretch | 2 | A′ |  | 2*A*_g_, 2*B*_2g_, 2*B*_1u_, 2*B*_3u_ |
| 4 C–H stretch | 1 | A″ |  | *B*_1g_, *B*_3g_, *A*_u_, *B*_2u_ |
| 4 C–H bend | 2 | A′ |  | 2*A*_g_, 2*B*_2g_, 2*B*_1u_, 2*B*_3u_ |
| 4 C–H bend | 1 | A″ |  | *B*_1g_, *B*_3g_, *A*_u_, *B*_2u_ |
| 4 Methyl rock | 1 | A′ |  | *A*_g_, *B*_2g_, *B*_1u_, *B*_3u_ |
| 4 Methyl rock | 1 | A″ |  | *B*_1g_, *B*_3g_, *A*_u_, *B*_2u_ |
| 4 S=O stretch | 2 | A′ |  | 2*A*_g_, 2*B*_2g_, 2*B*_1u_, 2*B*_3u_ |
| 4 S=O stretch | 1 | A″ |  | *B*_1g_, *B*_3g_, *A*_u_, *B*_2u_ |
| 4 S=O bend | 2 | A′ |  | 2*A*_g_, 2*B*_2g_, 2*B*_1u_, 2*B*_3u_ |
| 4 S=O bend | 1 | A″ |  | *B*_1g_, *B*_3g_, *A*_u_, *B*_2u_ |
| 4 C–S stretch | 1 | A′ |  | *A*_g_, *B*_2g_, *B*_1u_, *B*_3u_ |
| 4 Sulfonate rock | 1 | A′ |  | *A*_g_, *B*_2g_, *B*_1u_, *B*_3u_ |
| 4 Sulfonate rock | 1 | A″ |  | *B*_1g_, *B*_3g_, *A*_u_, *B*_2u_ |
| 4 C–S torsion | 1 | A″ |  | *B*_1g_, *B*_3g_, *A*_u_, *B*_2u_ |
|  |  |  |  |  |
| Acoustic modes |  |  |  | *B*_1u_, *B*_2u_, *B*_3u_ |

**Factor group splitting in a centrosymmetric system**

For a centrosymmetric molecule in the gas phase the rule of mutual exclusion (no coincidences in the infrared and Raman spectra) is strictly valid. In the solid state, this is only rigorously true for a centrosymmetric molecule in a crystal that crystallises in a centrosymmetric system and has only one molecule in the primitive cell. A classic example is K_2_[PtCl_6_], which crystallises in a cubic space group and the [PtCl_6_]^2-^ ion occupies an octahedral site [3]. For a centrosymmetric crystal with two molecules in the unit cell, the vibrations form in-phase and out-of-phase pairs, this is the factor group splitting and arises from interactions between the molecules. In the limit that the interaction is zero, the in-phase and out-of-phase pairs are accidentally degenerate. If one mode is Raman active and the other is infrared active, then even though it is a centrosymmetric system, the modes will occur at the same transition energy in both spectra. It is the degree of coupling between the species that determines the difference in the transition energies in the two forms of spectroscopy.

This is readily demonstrated computationally. Figure S1 shows a centrosymmetric cell, space group *P*mmm, containing two N_2_ molecules. By varying the lattice parameter *a*, the intermolecular distance and hence the coupling between the molecules is modified. Table S3 shows the results. At the closest distance of 2.5 Å the difference between the infrared and Raman active modes is ~15 cm^-1^, at the largest distance of 12.822 Å it is less than 10^-4^ cm^-1^.


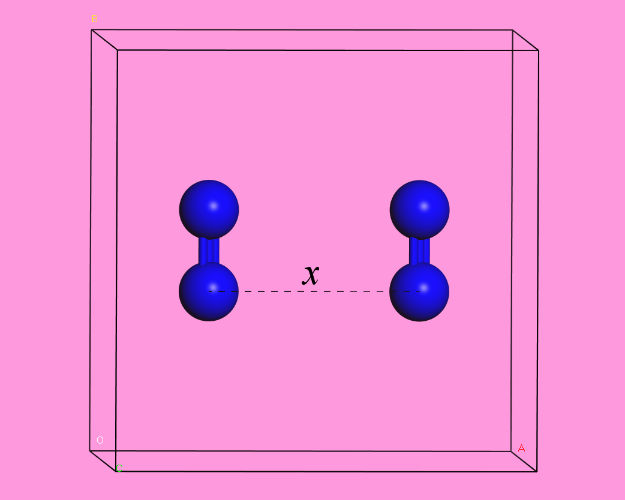


**Figure S1.** A hypothetical centrosymmetric cell, space group *P*mmm, containing two N_2_ molecules. *x* is the intermolecular distance.

**Table S3** Calculated infrared and Raman transition energies of N_2_ in a centrosymmetric crystal as a function of the intermolecular distance.

| Intermolecular distance, *x* / Å | Calculated transition energy / cm^-1^ | |  |
| --- | --- | --- | --- |
|  | Infrared | Raman | Difference |
| 2.500 | 2033.003096 | 2048.292885 | 15.289789 |
| 2.822 | 2032.568799 | 2038.430822 | 5.862023 |
| 7.822 | 2032.715915 | 2032.719082 | 0.003167 |
| 12.822 | 2032.726520 | 2032.726536 | 0.000016 |

**Table S4** Calculated modes with their assignments for Cs(CH_3_SO_3_)

| Transition energy  / cm^-1^ | Symmetry | Infrared intensity  / km mol^-1^ | Raman intensity  / Å^4^ amu^-1^ | Description |
| --- | --- | --- | --- | --- |
| 0 | *B*_2u_ | 0.00 | 0.00 | Acoustic mode |
| 0 | *B*_1u_ | 0.00 | 0.00 | Acoustic mode |
| 0 | *B*_3u_ | 0.00 | 0.00 | Acoustic mode |
| 31 | *A*_g_ | 0.00 | 0.17 | Translation |
| 38 | *B*_1g_ | 0.00 | 0.04 | Translation |
| 45 | *A*_u_ | 0.00 | 0.00 | Translation |
| 49 | *B*_2g_ | 0.00 | 0.00 | Translation |
| 49 | *B*_3u_ | 3.93 | 0.00 | Translation |
| 56 | *B*_3g_ | 0.00 | 0.20 | Translation |
| 59 | *A*_u_ | 0.00 | 0.00 | Libration |
| 59 | *B*_1u_ | 2.47 | 0.00 | Translation |
| 59 | *A*_g_ | 0.00 | 0.16 | Translation |
| 60 | *B*_2g_ | 0.00 | 0.00 | Translation |
| 65 | *B*_3u_ | 27.41 | 0.00 | Translation |
| 67 | *A*_g_ | 0.00 | 0.18 | Translation |
| 68 | *B*_3g_ | 0.00 | 0.02 | Libration |
| 68 | *B*_2u_ | 85.87 | 0.00 | Libration |
| 73 | *B*_1u_ | 65.75 | 0.00 | Translation |
| 73 | *B*_1g_ | 0.00 | 0.28 | Translation |
| 75 | *A*_g_ | 0.00 | 0.24 | Translation |
| 82 | *B*_2u_ | 22.94 | 0.00 | Translation |
| 84 | *B*_3g_ | 0.00 | 0.00 | Translation |
| 89 | *B*_2g_ | 0.00 | 0.05 | Translation |
| 89 | *A*_u_ | 0.00 | 0.00 | Libration |
| 90 | *B*_1u_ | 83.57 | 0.00 | Translation |
| 90 | *B*_3u_ | 115.79 | 0.00 | Translation |
| 96 | *A*_g_ | 0.00 | 2.45 | Libration |
| 97 | *B*_2u_ | 119.26 | 0.00 | Libration |
| 104 | *B*_3g_ | 0.00 | 1.03 | Libration |
| 104 | *B*_1g_ | 0.00 | 0.05 | Libration |
| 109 | *A*_u_ | 0.00 | 0.00 | Libration |
| 111 | *B*_2g_ | 0.00 | 0.05 | Translation |
| 122 | *B*_1g_ | 0.00 | 0.26 | Translation |
| 127 | *B*_3u_ | 53.90 | 0.00 | Libration |
| 139 | *B*_2g_ | 0.00 | 0.15 | Libration |
| 140 | *B*_1u_ | 26.39 | 0.00 | Libration |
| 257 | *B*_3g_ | 0.00 | 4.22 | C–S torsion (*A*_2_) |
| 258 | *B*_1g_ | 0.00 | 1.64 | C–S torsion (*A*_2_) |
| 264 | *A*_u_ | 0.00 | 0.00 | C–S torsion (*A*_2_) |
| 266 | *B*_2u_ | 7.19 | 0.00 | C–S torsion (*A*_2_) |
| 313 | *B*_2g_ | 0.00 | 1.09 | SO_3_ rock (*E*) |
| 317 | *B*_1u_ | 23.89 | 0.00 | SO_3_ rock (*E*) |
| 324 | *B*_3u_ | 29.40 | 0.00 | SO_3_ rock (*E*) |
| 328 | *A*_g_ | 0.00 | 26.91 | SO_3_ rock (*E*) |
| 329 | *A*_u_ | 0.00 | 0.00 | SO_3_ rock (*E*) |
| 331 | *B*_1g_ | 0.00 | 20.98 | SO_3_ rock (*E*) |
| 332 | *B*_2u_ | 14.04 | 0.00 | SO_3_ rock (*E*) |
| 337 | *B*_3g_ | 0.00 | 6.27 | SO_3_ rock (*E*) |
| 502 | *B*_1u_ | 55.81 | 0.00 | SO_3_ asymmetric bend (*E*) |
| 503 | *A*_g_ | 0.00 | 15.29 | SO_3_ asymmetric bend (*E*) |
| 504 | *B*_2g_ | 0.00 | 7.24 | SO_3_ asymmetric bend (*E*) |
| 505 | *B*_3u_ | 94.56 | 0.00 | SO_3_ asymmetric bend (*E*) |
| 508 | *B*_2u_ | 173.45 | 0.00 | SO_3_ asymmetric bend (*E*) |
| 509 | *B*_3g_ | 0.00 | 7.53 | SO_3_ asymmetric bend (*E*) |
| 510 | *B*_1g_ | 0.00 | 18.27 | SO_3_ asymmetric bend (*E*) |
| 510 | *A*_u_ | 0.00 | 0.00 | SO_3_ asymmetric bend (*E*) |
| 526 | *A*_g_ | 0.00 | 43.21 | SO_3_ symmetric bend (*A*_1_) + C–S stretch (*A*_1_) |
| 527 | *B*_3u_ | 116.04 | 0.00 | SO_3_ symmetric bend (*A*_1_) + C–S stretch (*A*_1_) |
| 529 | *B*_1u_ | 158.58 | 0.00 | SO_3_ symmetric bend (*A*_1_) + C–S stretch (*A*_1_) |
| 537 | *B*_2g_ | 0.00 | 36.55 | SO_3_ symmetric bend (*A*_1_) + C–S stretch (*A*_1_) |
| 735 | *B*_3u_ | 227.56 | 0.00 | C–S stretch (*A*_1_) + SO_3_ symmetric bend (*A*_1_) + |
| 736 | *A*_g_ | 0.00 | 181.71 | C–S stretch (*A*_1_) + SO_3_ symmetric bend (*A*_1_) + |
| 738 | *B*_1u_ | 267.22 | 0.00 | C–S stretch (*A*_1_) + SO_3_ symmetric bend (*A*_1_) + |
| 746 | *B*_2g_ | 0.00 | 14.81 | C–S stretch (*A*_1_) + SO_3_ symmetric bend (*A*_1_) + |
| 949 | *A*_g_ | 0.00 | 6.36 | CH_3_ rock (*E*) |
| 953 | *B*_2g_ | 0.00 | 0.06 | CH_3_ rock (*E*) |
| 956 | *B*_3u_ | 29.56 | 0.00 | CH_3_ rock (*E*) |
| 958 | *B*_1u_ | 0.00 | 0.00 | CH_3_ rock (*E*) |
| 967 | *B*_3g_ | 0.00 | 12.61 | CH_3_ rock (*E*) |
| 967 | *B*_1g_ | 0.00 | 0.07 | CH_3_ rock (*E*) |
| 971 | *B*_2u_ | 12.18 | 0.00 | CH_3_ rock (*E*) |
| 971 | *A*_u_ | 0.00 | 0.00 | CH_3_ rock (*E*) |
| 1005 | *B*_1u_ | 628.14 | 0.00 | SO_3_ symmetric stretch (*A*_1_) |
| 1005 | *A*_g_ | 0.00 | 484.04 | SO_3_ symmetric stretch (*A*_1_) |
| 1008 | *B*_3u_ | 451.62 | 0.00 | SO_3_ symmetric stretch (*A*_1_) |
| 1022 | *B*_2g_ | 0.00 | 64.15 | SO_3_ symmetric stretch (*A*_1_) |
| 1142 | *A*_g_ | 0.00 | 55.22 | SO_3_ asymmetric stretch (*E*) |
| 1155 | *B*_3u_ | 1650.52 | 0.00 | SO_3_ asymmetric stretch (*E*) |
| 1156 | *A*_u_ | 0.00 | 0.00 | SO_3_ asymmetric stretch (*E*) |
| 1156 | *B*_3g_ | 0.00 | 68.62 | SO_3_ asymmetric stretch (*E*) |
| 1158 | *B*_1g_ | 0.00 | 2.91 | SO_3_ asymmetric stretch (*E*) |
| 1158 | *B*_2u_ | 2683.77 | 0.00 | SO_3_ asymmetric stretch (*E*) |
| 1163 | *B*_1u_ | 1092.87 | 0.00 | SO_3_ asymmetric stretch (*E*) |
| 1200 | *B*_2g_ | 0.00 | 6.35 | SO_3_ asymmetric stretch (*E*) |
| 1294 | *A*_g_ | 0.00 | 8.91 | CH_3_ symmetric bend (*A*_1_) |
| 1298 | *B*_2g_ | 0.00 | 0.44 | CH_3_ symmetric bend (*A*_1_) |
| 1299 | *B*_3u_ | 68.16 | 0.00 | CH_3_ symmetric bend (*A*_1_) |
| 1299 | *B*_1u_ | 111.60 | 0.00 | CH_3_ symmetric bend (*A*_1_) |
| 1406 | *B*_2u_ | 13.12 | 0.00 | CH_3_ asymmetric bend (*E*) |
| 1411 | *A*_u_ | 0.00 | 0.00 | CH_3_ asymmetric bend (*E*) |
| 1418 | *B*_3u_ | 16.15 | 0.00 | CH_3_ asymmetric bend (*E*) |
| 1422 | *B*_1u_ | 18.00 | 0.00 | CH_3_ asymmetric bend (*E*) |
| 1424 | *B*_3g_ | 0.00 | 5.25 | CH_3_ asymmetric bend (*E*) |
| 1427 | *B*_1g_ | 0.00 | 71.71 | CH_3_ asymmetric bend (*E*) |
| 1430 | *A*_g_ | 0.00 | 65.64 | CH_3_ asymmetric bend (*E*) |
| 1433 | *B*_2g_ | 0.00 | 27.63 | CH_3_ asymmetric bend (*E*) |
| 2961 | *B*_1u_ | 0.06 | 0.00 | CH_3_ symmetric stretch (*A*_1_) |
| 2961 | *B*_3u_ | 1.24 | 0.00 | CH_3_ symmetric stretch (*A*_1_) |
| 2964 | *A*_g_ | 0.00 | 2889.85 | CH_3_ symmetric stretch (*A*_1_) |
| 2965 | *B*_2g_ | 0.00 | 91.89 | CH_3_ symmetric stretch (*A*_1_) |
| 3065 | *A*_u_ | 0.00 | 0.00 | CH_3_ asymmetric stretch (*E*) |
| 3065 | *B*_2u_ | 10.99 | 0.00 | CH_3_ asymmetric stretch (*E*) |
| 3067 | *B*_1g_ | 0.00 | 13.57 | CH_3_ asymmetric stretch (*E*) |
| 3067 | *B*_3g_ | 0.00 | 1011.72 | CH_3_ asymmetric stretch (*E*) |
| 3081 | *B*_2g_ | 0.00 | 69.13 | CH_3_ asymmetric stretch (*E*) |
| 3082 | *A*_g_ | 0.00 | 835.11 | CH_3_ asymmetric stretch (*E*) |
| 3082 | *B*_1u_ | 55.31 | 0.00 | CH_3_ asymmetric stretch (*E*) |
| 3082 | *B*_3u_ | 40.54 | 0.00 | CH_3_ asymmetric stretch (*E*) |





**Figure S2.** Comparison of experimental (purple) and calculated (blue) Raman spectra of Cs(CH_3_SO_3_).





**Figure S3.** Comparison of experimental (purple) and calculated (blue) infrared spectra of Cs(CH_3_SO_3_). The calculated spectrum uses a bandwidth of 20 cm^-1^, except for the 1050 – 1250 cm^-1^ region (cyan) where a bandwidth of 30 cm^-1^ is used in order to better match the experimental width in this region.





**Figure S4.** Comparison of experimental (purple) and calculated (blue) infrared spectra of Na(CH_3_SO_3_).

**

**

**Figure S5.** Infrared spectra, 0 – 4000 cm^-1^, of: (a) Cu(H_2_O)_4_(CH_3_SO_3_)_2_,
(b) Cu(D_2_O)_4_(CH_3_SO_3_)_2_, (c) Cd(H_2_O)_2_(CH_3_SO_3_)_2_, (d) Cd(D_2_O)_2_(CH_3_SO_3_)_2_ and (e) Ag(CH_3_SO_3_).

**References**

1. Brandon JK, Brown ID. 1967 Crystal structure of cesium methylsulfonate, CsCH_3_SO_3_. *Can. J. Chem*. **45**, 1385-1390. (doi:10.1139/v67-229).
2. Fateley WG, Dollish FR, McDevitt NT, Bentley FF. Infrared and Raman Selection Rules for Molecular and Lattice Vibrations: The Correlation Method, Wiley-Interscience, 1972.
3. Parker SF, Forsyth JB. 1998 K_2_MCl_6_ (M= Pt, Ir), location of the silent modes and force fields. *J. Chem. Soc., Faraday Trans.* **94** 1111-1114. (doi:10.1039/A709215F).
